# Health services utilization of Chinese patients with Huntington’s disease: a cross-sectional study

**DOI:** 10.1186/s12913-021-06826-1

**Published:** 2021-08-12

**Authors:** Huiyi Ke, Xi Cao, Yanyan Song, Li Cao

**Affiliations:** 1 Department of Neurology, Shanghai Jiao Tong University Affiliated Sixth People’s Hospital, Shanghai, China; 2 Department of Clinical Medicine, Shanghai Jiao Tong University School of Medicine, Shanghai, China; 3 Chinese Huntington’s Disease Association, Shanghai, China; 4 Department of Biostatistics, Institute of Medical Sciences, Shanghai Jiao Tong University School of Medicine, Shanghai, China; 5 Department of Neurology, Institute of Neurology, Rui Jin Hospital & Rui Jin Hospital North, Shanghai Jiao Tong University School of Medicine, Shanghai, China

**Keywords:** Huntington’s disease, Health services utilization, Andersen’s behavioral model, Follow-up medical visits

## Abstract

**Background:**

Huntington’s disease (HD) is a hereditary disease which could have a large impact on patients’ quality of life. As the neurodegenerative disorders progress, HD patients are expected to regularly take follow-up medical visits for proper treatment. This study aimed to analyze the general situation of health services utilization of Chinese HD patients and factors associated with their adherence to follow-up medical visits.

**Methods:**

We collected data from a questionnaire-based investigation conducted by the Chinese Huntington’s Disease Association. Data from 232 respondents were included to investigate whether they adhered to regular follow-up medical visits and the influencing factors. Based on Andersen’s behavioral model, the independent variables were categorized into predisposing, enabling and need factors. The variables were analyzed by chi-square test and stepwise logistic regression analysis.

**Results:**

Thirty-one point nine percent of the respondents had regular follow-up medical visits over the past year. Univariate analysis showed that there were significant differences with 6 factors (*P* < 0.05), among which, according to logistic regression, 2 enabling factors (reimbursement of health insurance, need for accompanying family members to follow-up visits) and 3 need factors (perceived stage of disease, perceived effectiveness of drugs, self-care ability) were independent influencing factors of follow-up medical behaviors of Chinese HD patients. The predisposing factors investigated here did not play a part in determining patients’ adherence to follow-up visits.

**Conclusions:**

Poor adherence to medical visits among Chinese HD patients is derived from multiple factors, including reimbursement of health insurance, perceived stage of disease and effectiveness of drugs, need for accompanying family members and self-care ability. To promote HD patients’ health services utilization, the improvement of the health insurance system, the enhancement of social support and the development of therapeutic approaches still have a long way to go.

**Supplementary Information:**

The online version contains supplementary material available at 10.1186/s12913-021-06826-1.

## Background

Huntington’s disease (HD) is an autosomal dominant inherited neurodegenerative disease characterized by cognitive, motor and psychiatric disorders. The onset of symptoms is generally in middle-age, followed by an irreversible progression of symptoms over 15–20 years until patients’ premature death [1].

The prevalence of HD varies globally. In the Western populations, 10.6–13.7 individuals per 100,000 are affected [2]. The prevalence rate is considered much lower in China. An epidemiological study in Taiwan estimated that the average annual incidence rate of HD was 0.1/100,000 [3]. Among East Asians, the prevalence of HD is similarly reported to be 0.1-1 per 100,000 [4]. However, the precise prevalence rate in mainland China is yet to be investigated.

HD has a profound effect on patients’ quality of life, which begins with the diagnosis of an affected parent. After the onset of symptoms, patients suffer from reduced functional capacity, including chorea, dystonia, memory loss, depression, etc. The progression of motor and cognitive decline eventually leads to the need for 24-h care [2]. As HD is a chronic neurodegenerative disease with a spectrum of symptoms, patients would require multidisciplinary interventions and long-term disease management. The optimal management relies on a combination of pharmacological and nonpharmacological interventions. In clinical practice, the antichoreic drug tetrabenazine and neuroleptics including olanzapine are commonly used for symptomatic treatment. In some cases, multidisciplinary interventions involving psychiatric treatment, speech and language therapy and nutritional guidance are advisable. Despite the lack of disease-modifying treatments, long-term medical interventions are essential to HD patients for monitoring disease progression and alleviating symptoms. The goal of regular follow-up medical visits consists in adapting treatments to the changing needs of the patients, preempting problems that would probably arise at the following stage of the disease and optimizing the quality of life. Compared with the control group, patients who had adhered to a long-term multidisciplinary rehabilitation intervention displayed significantly improved clinical outcomes, indicating the benefits of prolonged medical interventions for HD patients [5].

It is noteworthy that ongoing healthcare services correlate closely with increased medical expenditure and financial affordability could thus be a concern. A number of studies reach the conclusion that the economic burden of HD is substantial in the global context [6–8]. The total expenditure arising from follow-up medical visits includes not only direct outpatient medical costs but also costs of transportation and accommodation, lost labor productivity costs, etc. For these Chinese patients, healthcare costs can be partly covered by basic medical insurance for urban employees or new rural cooperative medical insurance, whereas the economic burden could be largely expanded for patients without any health insurance plan.

Health services utilization not only depends on individual characteristics, but also associates with public health policies and socioeconomic trends. However, evidence on the situation of health services utilization of HD patients is scarce, especially for China. Herein, this study aimed to determine the adherence to follow-up medical care among Chinese HD patients surveyed by the Chinese Huntington’s Disease Association. We used Andersen’s model, a theoretical basis broadly used to analyze determining factors of health services utilization, to examine correlates and predictors of follow-up medical visits among Chinese HD patients. Our results have significance in providing concrete statistics on patients’ use of healthcare services, determining the obstacles they might encounter in seeking medical services, and developing measures to ameliorate patients’ health-services-utilization experiences.

## Methods

### Data collection

This was a cross-sectional study, using data from a questionnaire survey oriented towards HD patients registered in the Chinese Huntington’s Disease Association. Founded in 2016, the Chinese Huntington’s Disease Association is the only charity organization of HD community in China. The organization provides medical support, health management and community support services for patients with HD, and aims to help them improve their quality of life. The members include currently about 800 HD families around China who have registered via an online platform, with 2.44 patients in each family on average.

In this survey, structured online questionnaire survey and telephone interviews were adopted, mainly the questionnaire survey. Members diagnosed with HD were eligible for this study. They were approached via chatting groups on social media and completed the online survey by smartphone or computer. From October to December 2019, information was gathered from a total of 236 patients from all provinces, municipalities and autonomous regions in China except for Hainan, Taiwan and Macao, which made them reasonable representatives of the HD population in China. A total of 232 respondents completed the questionnaire and provided valid information. The survey was not anonymous. Informed consent was attached to the online questionnaire.

### Research model and variables

**Fig. 1:**
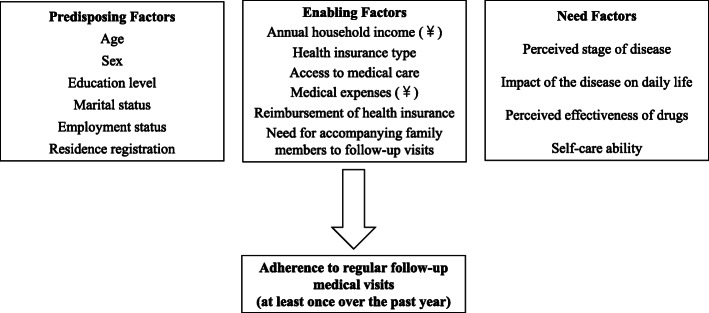
Research model and variables. Independent variables were categorized into predisposing, enabling and need factors. The dependent variable was whether the patient adhered to regular follow-up medical visits (at least once over the past year)

This study referred to Andersen’s behavioral model, which has been extensively applied in the investigation of health services utilization [9, 10]. This model explains the use of healthcare services by predisposing, enabling and need factors, into which the independent variables were categorized accordingly (Fig. 1).

### Study measures

Characteristics of interest in this study were investigated in the questionnaire in order to explore patients’ enabling and need factors associated with HD, as well as their demographic characteristics. The following predisposing factors were collected: age (8–17, 18–44, 45–64, ≥ 65 years), sex (male, female), education level (under elementary school, junior high school, senior high school, junior college, university or above), marital status (single, married, without children, married, with children, separated, divorced or widowed), employment status (employed, or enrolled student, retired, unemployed or unenrolled, unable to work or study), residence registration (urban, rural, according to China’s household registration system). Enabling factors included annual household income (¥, < 50,000, 50,000-149,999, ≥ 150,000, including total labor income, pension, benefits and financial assistance), health insurance type (basic medical insurance for urban employees, new rural cooperative medical insurance, without health insurance, according to China’s health insurance system), access to follow-up medical care (whether the patient received healthcare services at local hospitals, or had to travel across the city or the province), annual medical expenses related to HD treatments (¥, < 10,000, 10,000–49,999, ≥ 50,000, including direct medical costs, costs of transportation and accommodation and lost labor productivity costs, for the patient and the accompanying family members), whether the patient received reimbursement from the health insurance plan or not, need for accompanying family members to follow-up visits (no, 1 accompanying family member, 2 or more accompanying family members). Need factors were composed of several self-assessed variables related to the disease or the treatment, including perceived stage of disease (early stage, middle stage, late stage, unaware), impact of the disease on daily life (no impact, mild, moderate, severe), perceived effectiveness of drugs (effective, not very effective, not effective, unaware), self-care ability (completely able, able for the majority, able for the minority, completely unable). As several respondents had refused or failed to provide exact answers to certain questions, these answers had to be filtered. Thus, data missing existed for the following variables: employment status, annual household income, health insurance type, access to medical care, medical expenses, reimbursement of health insurance (For details see Table 1).

**Table 1:** Baseline characteristics and univariate analysis of adherence to follow-up visits in HD patients

Characteristic	N (%)	Adherent to follow-up visits	Non-adherent to follow-up visits	χ2	*P*
Predisposing factors					
Age				4.011	0.260
8-17	6 (2.6)	3 (1.3)	3 (1.3)		
18-44	80 (34.5)	31 (13.4)	49 (21.1)		
45-64	125 (53.9)	34 (14.7)	91 (39.2)		
≥65	21 (9.0)	6 (2.6)	15 (6.5)		
Sex				0.276	0.599
Male	103 (44.4)	31 (13.4)	72 (31.0)		
Female	129 (55.6)	43 (18.5)	86 (37.1)		
Education level				7.237	0.124
Under elementary school	45 (19.4)	13 (5.6)	32 (13.8)		
Junior high school	72 (31.0)	27 (11.6)	45 (19.4)		
Senior high school	50 (21.6)	10 (4.3)	40 (17.2)		
Junior college	39 (16.8)	17 (7.3)	22 (9.5)		
University or above	26 (11.2)	7 (3.0)	19 (8.2)		
Marital status				1.271	0.736
Single	23 (9.9)	6 (2.6)	17 (7.3)		
Married, without children	12 (5.2)	4 (1.7)	8 (3.4)		
Married, with children	164 (70.7)	51 (22.0)	113 (48.7)		
Separated, divorced or widowed	33 (14.2)	13 (5.6)	20 (8.6)		
Employment status				4.993	0.172
Employed, or enrolled student	41 (17.7)	11 (4.7)	30 (12.9)		
Retired	46 (19.8)	17 (7.3)	29 (12.5)		
Unemployed, or unenrolled	58 (25.0)	24 (10.3)	34 (14.7)		
Unable to work or study	86 (37.1)	22 (9.5)	64 (27.6)		
Residence registration				0.053	0.819
Urban	126 (54.3)	41 (17.7)	85 (36.6)		
Rural	106 (45.7)	33 (14.2)	73 (31.5)		
Enabling Factors					
Annual household income (¥)				3.063	0.216
<50,000	116 (50.0)	31 (13.4)	85 (36.6)		
50,000-149,999	89 (38.4)	34 (14.7)	55 (23.7)		
≥150,000	25 (10.8)	8 (3.4)	17 (7.3)		
Health insurance type				0.507	0.776
Basic medical insurance for urban employees	110 (47.4)	38 (16.4)	72 (31.0)		
New rural cooperative medical insurance	87 (37.5)	26 (11.2)	61 (26.3)		
Without health insurance	29 (12.5)	9 (3.9)	20 (8.6)		
Access to medical care				0.567	0.753
Local hospitals	77 (33.2)	30 (12.9)	47 (20.3)		
Cross-city	43 (18.5)	14 (6.0)	29 (12.5)		
Cross-province	67 (28.9)	26 (11.2)	41 (17.7)		
Medical expenses(¥)				8.824	0.012
<10,000	105 (45.3)	24 (10.3)	81 (34.9)		
10,000-49,999	88 (37.9)	35 (15.1)	53 (22.8)		
≥50,000	31 (13.4)	14 (6.0)	17 (7.3)		
Reimbursement of health insurance				10.22	0.001
Yes	51 (22.0)	26 (11.2)	25 (10.8)		
No	170 (73.3)	46 (19.8)	124 (53.4)		
Need for accompanying family members to follow-up visits				6.735	0.034
No	28 (12.1)	8 (3.4)	20 (8.6)		
1 accompanying family member	110 (47.4)	43 (18.5)	67 (28.9)		
2 or more accompanying family members	94 (40.5)	21 (9.1)	73 (31.5)		
Need factors					
Perceived stage of disease				15.701	0.001
Early stage	48 (20.7)	20 (8.6)	28 (12.1)		
Middle stage	101 (43.5)	41 (17.7)	60 (25.9)		
Late stage	52 (22.4)	8 (3.4)	44 (19.0)		
Unaware	31 (13.4)	5 (2.2)	26 (11.2)		
Impact of the disease on daily life				4.324	0.229
No	13 (5.6)	3 (1.3)	10 (4.3)		
Mild	32 (13.8)	8 (3.4)	24 (10.3)		
Moderate	51 (22.0)	22 (9.5)	29 (12.5)		
Severe	136 (58.6)	41 (17.7)	95 (40.9)		
Perceived effectiveness of drugs				37.71	<0.001
Effective	27 (11.6)	18 (7.8)	9 (3.9)		
Not very effective	77 (33.2)	34 (14.7)	43 (18.5)		
Not effective	66 (28.4)	17 (7.3)	49 (21.1)		
Unaware	62 (26.7)	5 (2.2)	57 (24.6)		
Self-care ability				8.635	0.035
Completely able	63 (27.2)	16 (6.9)	47 (20.3)		
Able for the majority	49 (21.1)	23 (9.9)	26 (11.2)		
Able for the minority	74 (31.9)	15 (6.5)	59 (25.4)		
Completely unable	46 (19.8)	10 (4.3)	36 (15.5)		

Data are presented as n (%). Data missing existed for employment status, annual household income, health insurance type, access to medical care, medical expenses, reimbursement of health insurance

The utilization of health services, the dependent variable in this study, was based on the question “How often have you had follow-up medical visits over the past year?”. Patients who reported to have taken follow-up medical visits at least once over the past year were considered to have adhered to regular follow-up visits. In clinical practice, the frequency of follow-up varies with the stage of HD, but annual visits are generally a minimum requirement. Patients who had less frequent follow-up were thus considered non-adherent to follow-up visits.

### Statistical method

The characteristics of the individuals were summarized by descriptive analysis and the results were presented as absolute frequencies and percentages. To identify the influencing factors of patients’ adherence to follow-up visits, univariate analysis and logistic regression analysis were used successively. The former was performed with chi-square test to examine the impacts of different variables. Logistic regression analysis with backward selection was further conducted to determine independent influencing factors (entry criterion = 0.05, removal criterion = 0.10). All analyses were performed with SPSS version 17.

## Results

Among the 232 respondents, 74 (31.9 %) had regular follow-up medical visits, and 158 (68.1 %) didn’t seek healthcare over the past year. As shown in Table 1, 53.9 % were middle-aged (between 45 and 64). Over half (55.6 %) were female, 70.7 % were currently married and had children, 37.1 % were unable to work or attend school, and urban residents accounted for 54.3 %. The median annual household income was approximately ¥50,000.

Comparing the patients who adhered to regular follow-up visits with those who didn’t, univariate analysis demonstrated that there were significant differences with the following 6 factors (*P* < 0.05): medical expenses, reimbursement of health insurance, need for accompanying family members to follow-up visits, perceived stage of disease, perceived effectiveness of drugs, self-care ability (Table 1). On the other hand, none of the predisposing factors (age, sex, education level, marital status, employment status, residence registration) showed significant differences (*P* > 0.05), nor did annual household income, health insurance type, access to medical care, or impact of the disease on daily life (Table 1). These results suggested that between the two groups of patients, disparities existed for the 6 aforementioned enabling or need factors, but not the others.

Stepwise multivariate logistic regression analysis was then conducted based on all the variables investigated (definition of the variables in the Methods section and Additional file 1). Hosmer and Lemeshow test verified good fitness of the model (*P* > 0.05). 5 variables remained in the model after backward selection, including reimbursement of health insurance, need for accompanying family members to follow-up visits, perceived stage of disease, perceived effectiveness of drugs, and self-care ability (*P* < 0.05, Table 2), revealing that these were the independent influencing factors of follow-up medical behaviors of HD patients. Specifically, with the reimbursement of health insurance for their medical costs, the patients were inclined to adhere to follow-up medical visits (OR = 3.276). The patients who needed only 1 accompanying family member for their medical visits were more likely to regularly visit their doctors than those who required 2 or more accompanying family members (OR = 3.488). Moreover, compared with the patients who believed themselves to be at the late stage of the disease, those self-estimated to be at the early or the middle stage tended to stick to follow-up visits (OR = 13.664, 3.746, respectively). Compared with the patients who considered the drugs ineffective, those unaware of whether the drugs were effective or not were even less likely to take follow-up visits (OR = 0.157). Besides, the patients who were partly able to take care of themselves adhered more to follow-up visits than those who were completely able (OR = 6.784, 4.632, for “Able for the majority” and “Able for the minority” respectively).

**Table 2:** Stepwise multivariate logistic regression analysis of adherence to follow-up visits in HD patients

Variables	β	OR	95 %CI	*P*
Coefficient	-4.012	0.018	-	< 0.001
Reimbursement of health insurance (No)				
Yes	1.187	3.276	(1.353, 7.929)	0.009
Need for accompanying family members to follow-up visits (2 or more accompanying family members)				
No	1.374	3.950	(0.879, 17.745)	0.073
1 accompanying family member	1.249	3.488	(1.545, 7.875)	0.003
Perceived stage of disease (Late stage)				
Unaware	0.989	2.688	(0.478, 15.118)	0.262
Early stage	2.615	13.664	(2.584, 72.246)	0.002
Middle stage	1.321	3.746	(1.174, 11.954)	0.026
Perceived effectiveness of drugs (Not effective)				
Unaware	-1.849	0.157	(0.040, 0.617)	0.008
Not very effective	0.313	1.368	(0.550, 3.403)	0.501
Effective	0.657	1.930	(0.593, 6.281)	0.275
Self-care ability (Completely able)				
Able for the majority	1.915	6.784	(1.750, 26.292)	0.006
Able for the minority	1.533	4.632	(1.123, 19.110)	0.034
Completely unable	1.166	3.210	(0.604, 17.068)	0.171

*OR* odds ratio, *CI* confidence interval

## Discussion

This is the first study to our knowledge to investigate the situation of health services utilization of Chinese HD patients and to analyze the determinants. Our initial finding was that less than 1/3 of the respondents took regular follow-up medical visits over the past year, suggesting extensive non-adherence to follow-up among HD patients. The discrepancy between the needs for healthcare and the utilization of health services among HD patients seems to be a global concern [11–14]. In the US, 42 % of HD patients received long-term care services including different types of healthcare services [15], while in this study, 68.1 % of the Chinese patients with HD did not access care over the past year, not to mention the adherence to ongoing healthcare. As suggested by the World Health Organization, improvement of adherence interventions may be far more efficient to promote population health than advances in biomedical treatments [16]. Failure to adhere to follow-up treatments could accelerate disease progression and eventually increase the economic burden [5, 8].

To enhance adherence, it is necessary to examine the origins of loss of follow-up. By logistic regression, we determined 2 enabling factors (reimbursement of health insurance, need for accompanying family members to follow-up visits) and 3 need factors (perceived stage of disease, perceived effectiveness of drugs, self-care ability) as the main factors influencing HD follow-up practices. The predisposing factors, namely the demographic characteristics of patients, were not among the correlates of health services utilization, which indicated that regardless of age, sex, education, marital and employment status, residence, Chinese HD patients generally had poor adherence to follow-up medical visits.

The reimbursement of health insurance largely promoted patients’ health services utilization, with an OR of 3.276 (Table 2). With substantial hospital services and drug costs, HD burdens patients with significant medical expenses in the long run [8]. The mean or average annual cost per patient with HD was reported to reach $8,120 in Peru [7], $3,257 for Medicaid patients and $4,947 for commercial patients in the US [8], and £21,605 in the UK [6]. In our study, more than half of the respondents spent >¥10,000 on medical intervention annually. However, up to 73.3 % of the patients claimed that they did not enjoy health insurance for their medical expenses (Table 1). Indeed, basic medical insurance failed to improve the affordability of medical costs of Chinese patients with rare diseases [17]. China is still exploring policies regarding the health insurance system for rare diseases. The antichoreic drug for HD, deutetrabenazine, is now covered by state-paid medical insurance plans. However, problems persist as patients from remote areas have to disburse cross-provincial medical costs and seek reimbursement afterward. Thus, the unavailability of reimbursement of health insurance, as well as the economic burden of the disease, could partly impede regular healthcare utilization of patients.

Besides, the patients self-assessed to be at the late stage of HD, as well as those who were not convinced of the effectiveness of drugs, were less likely to take follow-up visits. Previous studies showed that patients with poorer self-rated mental health were significantly more likely to receive treatment for emotional problems [18], which was not the case with the HD patients in our study. Faced with the gradual progression of disease and limited approach of treatment, the patients who were pessimistic or lost faith in the possibility to be cured could be less willing to comply with HD long-term management. Our examination of the need factors revealed how patients’ view of their health status and the cure played a part in determining their adherence to follow-up visits.

Other influencing factors of patients’ health services utilization included their need for accompanying family members to follow-up visits and self-care ability. We speculated that as the motor disorders progressively impaired patients’ self-care ability, they might sense the urgent need to seek medical care for the alleviation of symptoms. On the other hand, when a patient needs to be accompanied by 2 or more family members, follow-up visits may be rather troublesome and thus the frequency of medical visits declines.

Based on the research findings, we propose that to promote follow-up medical visits among HD patients, the availability and the coverage of basic health insurance plans should first be improved for rare diseases such as HD. Second, social and community support plays an essential part in long-term disease management. Both the patients and the caregivers bear a great burden in families with HD patients. As an invaluable source of information, mutual assistance, emotional support, or even financial aid, patient groups gather widely dispersed patients together and help patients and families through difficult times, encouraging adherence and compliance to disease management. Development of community-based care has also a significant potential for facilitating patients’ access to medical care and reducing the care burden for their families. These services highly warranted by HD patients remain a need that is currently unmet [14]. Third, despite research advances over the past two decades and the emergence of therapeutic strategies and clinical trials, there has unfortunately been minimal success to date and few approaches are available for HD treatment. As the shortage of therapeutic means was shown to contribute to the low utilization rate of health services among patients with rare diseases in China [19], the development of drugs and new therapeutic approaches is necessary. Nevertheless, it is important to realize that without the promotion of proper utilization of health services, new therapeutic approaches would probably be of little help to HD patients.

This study has the following limitations. First, due to the cross-sectional nature of the study, the causality or temporal relation between the frequency of healthcare utilization and the variables could not be determined. Second, due to the limitation of the investigation, the variables that we have included in this study may be incomplete. Third, the data were collected by questionnaire survey in which information was self-reported by patients and was subject to recall errors. Finally, data for some independent variables (employment status, annual household income, health insurance type, access to medical care, medical expenses, reimbursement of health insurance) were incomplete. Despite these limitations, our results highlight the determining role that enabling and need factors play in health services utilization of Chinese HD patients by applying Andersen’s model. This study also has significance in that it provides baseline data of one specific type of rare disease in China, and may serve as a reference for policy-making. The Penchansky framework provides future research with a helpful model for conceptualizing access to healthcare. The theory contains five dimensions: availability, accessibility, accommodation, affordability and acceptability [20]. Our study has taken into account the factors of affordability, accessibility and accommodation, while a full view of access to healthcare services of HD patients awaits further investigation.

## Conclusions

This study analyzed the situation of health services utilization of Chinese HD patients and the associated factors, using data from the Chinese Huntington’s Disease Association and Andersen’s behavioral model as theoretical basis. Our results suggested that non-adherence to follow-up medical visits was frequent among Chinese HD patients. Reimbursement of health insurance, need for accompanying family members to follow-up visits, perceived stage of disease, perceived effectiveness of drugs, and self-care ability were the 5 independent influencing factors of their follow-up medical behaviors. Therefore, measures to improve the medical practice and the quality of life of HD patients should focus on a more developed health insurance system, broader social support for rare disease sufferers, and researches for more effective treatments.

## Supplementary Information

**Additional file 1.** Definition of the variables for multivariate logistic regression analysis.

## Data Availability

The datasets used and/or analyzed during the current study are available from the corresponding author on reasonable request.

## Abbreviations

HD: Huntington’s Disease
OR: Odds ratio
S.E.: standard error
CI: Confidence Interval
