# Supplementary material for: Health services utilization of Chinese patients with Huntington’s disease: a cross-sectional study
Source: BMC Health Serv Res. 2021 Aug 12;21:806. doi: 10.1186/s12913-021-06826-1 (PMC8362235; doi:10.1186/s12913-021-06826-1)
Supplement: Supplementary file 1 — Additional file 1. Definition of the variables for multivariate logistic regression analysis. [file 12913_2021_6826_MOESM1_ESM.docx]

**Additional file 1** Definition of the variables for multivariate logistic regression analysis

| Variable | Definition |
| --- | --- |
| Adherence to regular follow-up medical visits over the past year | No=0, Yes=1 |
| Age | 8-17=1, 18-44=2, 45-64=3, ≥65=4 |
| Gender | Male=1, Female=2 |
| Education level | Under elementary school=1, Junior high school=2, Senior high school=3, Junior college=4, University or above=5 |
| Marital status | Single=1, Married, without children=2, Married, with children=3, Separated, divorced or widowed=4 |
| Employment status | Employed, or enrolled student=1, Retired=2, Unemployed, or unenrolled=3, Disable to work or study=4 |
| Residence registration | Urban=1, Rural=2 |
| Annual household income (￥) | <50,000=1, 50,000-149,999=2, ≥150,000=3 |
| Health insurance type | Basic medical insurance for urban employees=1, New rural cooperative medical insurance=2, Without health insurance=3 |
| Access to medical care | Local hospitals=1, Cross-city=2, Cross-province=3 |
| Reimbursement of health insurance | No=0, Yes=1 |
| Medical expenses(￥) | <10,000=1, 10,000~49,999=2, ≥50,000=3 |
| Need for accompanying family members to follow-up visits | No=1, 1 accompanying family member=2, 2 or more accompanying family members=3 |
| Perceived stage of disease | Late stage=1, Early stage=2, Middle stage=3, Unaware=4 |
| Impact of the disease on daily life | No=1, Mild=2, Moderate=3, Severe=4 |
| Perceived effectiveness of drugs | Not effective=1, Unaware=2, Not very effective=3, Effective=4 |
| Self-care ability | Completely able=1, Able for the majority=2, Able for the minority=3, Completely unable=4 |
